# Outcomes of selective nonoperative management of civilian abdominal gunshot wounds: a systematic review and meta-analysis

**DOI:** 10.1186/s13017-018-0215-0

**Published:** 2018-11-27

**Authors:** Aziza N. Al Rawahi, Fatma A. Al Hinai, Jamie M. Boyd, Christopher J. Doig, Chad G. Ball, George C. Velmahos, Andrew W. Kirkpatrick, Pradeep H. Navsaria, Derek J. Roberts

**Affiliations:** 1 Department of Surgery, University of Calgary, Calgary, Alberta Canada; 2 Department of Critical Care Medicine, University of Calgary, Calgary, Alberta Canada; 3 Regional Trauma Program, University of Calgary and the Foothills Medical Centre, Calgary, Alberta Canada; 4 Division of Trauma, Emergency Surgery, and Critical Care, Department of Surgery, Massachusetts General Hospital, Boston, MA USA; 5 Department of Surgery, University of Cape Town Health Sciences Faculty, Cape Town, South Africa; 6 Trauma Centre, Groote Schuur Hospital, Observatory, Cape Town, South Africa; 7 Division of Vascular and Endovascular Surgery, Department of Surgery, University of Ottawa, The Ottawa Hospital, Civic Campus, Room A280, 1053 Carling Avenue, Ottawa, Ontario K1Y 4E9 Canada

**Keywords:** Abdominal gunshot wounds, Selective nonoperative management, Penetrating trauma, Wounds and injuries

## Abstract

**Background:**

Although mandatory laparotomy has been standard of care for patients with abdominal gunshot wounds (GSWs) for decades, this approach is associated with non-therapeutic operations, morbidity, and long hospital stays. This systematic review and meta-analysis sought to summarize outcomes of selective nonoperative management (SNOM) of civilian abdominal GSWs.

**Methods:**

We searched electronic databases (March 1966–April 1, 2017) and reference lists of articles included in the systematic review for studies reporting outcomes of SNOM of civilian abdominal GSWs. We meta-analyzed the associated risks of SNOM-related failure (defined as laparotomy during hospital admission), mortality, and morbidity across included studies using DerSimonian and Laird random-effects models. Between-study heterogeneity was assessed by calculating *I*^2^ statistics and conducting tests of homogeneity.

**Results:**

Of 7155 citations identified, we included 41 studies [*n* = 22,847 patients with abdominal GSWs, of whom 6777 (29.7%) underwent SNOM]. The pooled risk of failure of SNOM in hemodynamically stable patients without a reduced level of consciousness or signs of peritonitis was 7.0% [95% confidence interval (CI) = 3.9–10.1%; *I*^2^ = 92.6%, homogeneity *p* < 0.001] while the pooled mortality associated with use of SNOM in this patient population was 0.4% (95% CI = 0.2–0.6%; *I*^2^ = 0%, homogeneity *p* > 0.99). In patients who failed SNOM, the pooled estimate of the risk of therapeutic laparotomy was 68.0% (95% CI = 58.3–77.7%; *I*^2^ = 91.5%; homogeneity *p* < 0.001). Risks of failure of SNOM were lowest in studies that evaluated patients with right thoracoabdomen (3.4%; 95% CI = 0–7.0%; *I*^2^ = 0%; homogeneity *p* = 0.45), flank (7.0%; 95% CI = 3.9–10.1%), and back (3.1%; 95% CI = 0–6.5%) GSWs and highest in those that evaluated patients with anterior abdomen (13.2%; 95% CI = 6.3–20.1%) GSWs. In patients who underwent mandatory abdominopelvic computed tomography (CT), the pooled risk of failure was 4.1% versus 8.3% in those who underwent selective CT (*p* = 0.08). The overall sample-size-weighted mean hospital length of stay among patients who underwent SNOM was 6 days versus 10 days if they failed SNOM or developed an in-hospital complication.

**Conclusions:**

SNOM of abdominal GSWs is safe when conducted in hemodynamically stable patients without a reduced level of consciousness or signs of peritonitis. Failure of SNOM may be lower in patients with GSWs to the back, flank, or right thoracoabdomen and be decreased by mandatory use of abdominopelvic CT scans.

**Electronic supplementary material:**

The online version of this article (10.1186/s13017-018-0215-0) contains supplementary material, which is available to authorized users.

## Background

Mandatory laparotomy has been standard of care for patients with abdominal gunshot wounds (GSWs) for decades. However, this approach is associated with unnecessary, including non-therapeutic (where intra-abdominal injuries are found that do not require intervention) and negative (where no intra-abdominal injuries are found), laparotomies [1–3]. Further, mandatory laparotomy in patients with abdominal GSWs has been linked with a 22–41% risk of postoperative complications (e.g., surgical site infections, gastrointestinal ileus, pneumonia, and venous thromboembolism) [4, 5] and a 5- to 9-day length of hospital stay [6–8].

Selective nonoperative management (SNOM) is frequently conducted in trauma centers in patients with penetrating abdominal trauma who are hemodynamically stable without signs of diffuse peritonitis or evisceration. Although SNOM has been relatively widely adopted for abdominal stab wounds [1, 3, 9], the concept has not been as embraced for GSWs given the higher associated incidence of visceral and abdominal vascular injuries and the morbidity and mortality associated with missed injuries [9, 10]. Therefore, the practice of SNOM for abdominal GSWs remains controversial among some surgeons.

The cornerstone of SNOM lies on the principle of serial physical examinations of patients without a reduced level of consciousness by qualified surgeons or experienced surgical residents. Over the last two decades, there has also been an interest in using abdominopelvic computed tomography (CT) scans as an adjunct when conducting SNOM [11, 12]. Proponents suggest that CT scans may better characterize bullet trajectory and have been reported to detect injuries with a sensitivity and specificity exceeding 90% [12]. Opponents argue that it is associated with false negative and positive test results and lacks accuracy in detecting some (e.g., intestinal and diaphragmatic) injuries [13].

There have been no randomized controlled trials to date that have evaluated the outcomes of SNOM versus mandatory laparotomy for management of civilian abdominal GSWs. Moreover, results of cohort studies of this injury management strategy have been variable. To evaluate the potential safety of this approach, the purpose of this systematic review and meta-analysis was to summarize outcomes associated with use of SNOM in cohort studies of civilians with abdominal GSWs. We hypothesized that SNOM of abdominal GSWs would be safe (associated with a small risk of failure, morbidity, and mortality) when conducted in highly experienced trauma centers. We also sought to determine whether variability in reported outcomes of SNOM across cohort studies may be due to differences in study risks of bias, practices of SNOM across trauma centers and countries, or study patient injury patterns (e.g., whether the entry wound of the included patients was predominantly in the anterior abdomen, thoracoabdomen, flank, or back).

## Methods

### Protocol

Our methods were pre-specified in a detailed protocol created according to the Preferred Reporting Items in Systematic Reviews and Meta-Analyses statement [14] and the Meta-analysis of Observational Studies in Epidemiology (MOOSE) proposal (see the completed MOOSE checklist in Additional file 1: digital content S1) [15].

### Search strategy

With the assistance of a medical librarian/information scientist, we searched PubMed, Ovid MEDLINE and EMBASE, and the Cochrane Library from their inception to April 1, 2017, without language restrictions. Using a combination of Medical Subject Heading (MeSH) and Emtree terms and keywords, we created search filters covering the themes SNOM, penetrating injury/GSW, and abdomen. These filters were combined in our final database searches using the Boolean operator “AND.” Our complete search strategies are shown in Additional file 2: digital content S2. To identify additional studies, we used the PubMed “related articles” feature, hand-searched references of included original and relevant review articles identified during the search, and wrote several authors who had published on the topic.

### Selection criteria

Independently and in duplicate, two investigators (ANAR, FAAH) screened the titles and abstracts of citations identified during the search, reviewed potentially relevant articles in full, and decided on study inclusion. We used the following inclusion criteria: (1) study participants were adult (mean age ≥ 16 years) civilian trauma patients with GSWs to the abdomen; (2) some or all of the included patients underwent SNOM of their abdominal GSWs; (3) reported outcomes included SNOM failure, morbidity, mortality, and/or patient hospital length of stay (LOS); and (4) the study used a cohort design. We distinguished cohort studies from case series using the criteria developed by Dekkers et al. and included controlled (which included a comparable control group of patients without diffuse peritonitis or hemodynamic instability that underwent mandatory laparotomy) and uncontrolled cohort studies in the systematic review [16]. We excluded conference abstracts. Study eligibility disagreements were resolved by consensus. Inter-investigator agreement on article inclusion was assessed using kappa (κ) statistics [17]. When the same data were reported across multiple studies, the study with the larger sample size or that provided the most information on SNOM-associated outcomes was included.

### Definitions

We subdivided the abdomen into the anterior abdomen, thoracoabdomen, bilateral flanks, and back. We defined the anterior abdomen as the region bounded superiorly by the costal margins, laterally by the anterior axillary lines, and inferiorly by the inguinal creases [18]. The thoracoabdomen was defined as the region enclosed by the nipples (or tips of the scapulae) superiorly and the costal margin inferiorly [18]. We defined the flanks as the region bounded by the costal margins, anterior and posterior axillary lines, and iliac crests [18]. Finally, the back was defined as the region bounded superiorly by the inferior scapular tips, laterally by the posterior axillary lines, and inferiorly by the iliac crests [18].

### Data extraction

Three reviewers (ANAR, FAAH, JMB) independently extracted data from included studies in duplicate. Data extracted included (1) study design, temporality, and setting; (2) trauma center and study cohort characteristics [e.g., recruitment period and mean/median patient age and Injury Severity Scale (ISS) score], number of trauma patients and abdominal GSWs assessed per year, and the anatomical regions of the abdomen injured by GSWs; (3) number of patients who underwent SNOM and details regarding SNOM practices (frequency of clinical and laboratory examinations, mandatory versus selective use of CT, duration of observation, and type and duration of follow-up); and (4) outcomes associated with use of SNOM.

### Primary and secondary outcomes

The primary outcome was the risk of failure of SNOM. We defined failure as the conduct of laparotomy on a patient undergoing SNOM for an abdominal GSW during their hospital admission. Secondary outcomes included therapeutic and unnecessary laparotomy among patients who failed SNOM, in-hospital mortality, reported morbidities associated with SNOM, and hospital LOS. Unnecessary laparotomy was defined as either negative (where no injury was identified during laparotomy) or non-therapeutic (where an injury was found during laparotomy that did not require surgical intervention).

### Risk of bias assessment

The risk of bias of the included studies was assessed by two independent investigators with graduate training in epidemiology (ANAR, FAAH) using a modified version of the Quality in Prognosis Studies (QUIPS) tool [19]. This tool includes 24 decision items that cover five quality domains of interest, including (1) patient selection, (2) study attrition, (3) prognostic factor measurement, (4) outcome measurement, and (5) statistical analysis and reporting. We scored the adequacy of reporting for each item of the above five QUIPS domains as “yes,” “no,” or “unclear.” This scoring led to the overall judgment of low, moderate, or high risk of bias per quality domain. Disagreements regarding study risk of bias were resolved by consensus.

### Statistical analyses

We calculated study estimates of the risk of failure of SNOM and therapeutic and unnecessary laparotomy, reported morbidities, and in-hospital mortality associated with use of SNOM in patients with abdominal GSWs. We determined standard errors and 95% confidence intervals (CIs) for these estimates using Clopper-Pearson exact methods [20]. We applied a continuity correction of 1 to estimates with a zero numerator or denominator to estimate their standard error [21].

DerSimonian and Laird random-effects models were used to calculate pooled estimates of the risk of outcomes across the included studies [22]. Summary mean and median LOS across studies were calculated by weighting these estimates by study sample sizes. Heterogeneity in pooled estimates was assessed by calculating *I*^2^ inconsistency and Q statistics and conducting tests of homogeneity (*p* value < 0.10 considered significant given the low power of these tests) [23]. The *I*^2^ statistic represents the percentage of variation between studies due to factors other than chance. *I*^2^ statistics of > 25%, > 50%, and > 75% were considered to represent low, moderate, and high degrees of heterogeneity, respectively [24]. A test of homogeneity *p* value < 0.10 was considered to indicate more heterogeneity than would be expected between studies due solely to chance [24].

In the presence of at least low inter-study heterogeneity, we conducted stratified meta-analyses and meta-regression to determine whether our pooled risk estimates varied across a number of study-level covariates selected a priori. Covariates of interest included study setting (USA, South Africa, or other) and temporality (prospective versus retrospective), study patient injury patterns [anterior abdominal, right thoracoabdominal, isolated renal or hepatic/right upper quadrant (RUQ), back, or flank GSWs], and reported SNOM practices (serial physical examinations done by surgeons or surgical residents and mandatory versus selective use of abdominopelvic CT scans).

We examined for evidence of small study effects potentially due to publication bias by creating funnel plots and conducting Begg’s funnel plot asymmetry test [25]. We used the Duval and Tweedie “trim and fill” method to evaluate the potential influence of publication bias on our pooled estimates [26, 27]. Using this method, small outlying studies were first “trimmed” (removed until funnel plots were symmetric). The remaining study results were then used to re-estimate the theoretically unbiased center of the plot before it was “filled” (the missing outlying study results and their theoretical counterparts were replaced around the re-estimated center), permitting calculation of a publication bias-adjusted pooled risk estimate [26–29]. Stata version 13 (Stata Corp., College Station, TX, USA) was used for all analyses.

## Results

### Study selection

**Fig. 1:**
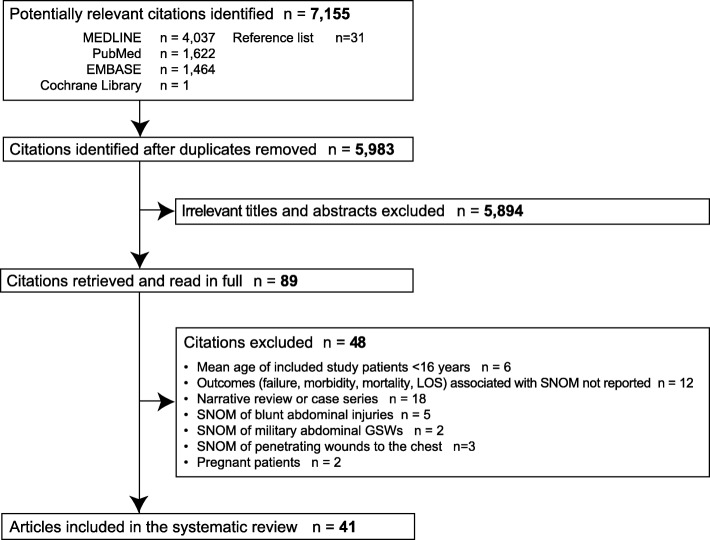
Flow of articles through the systematic review, where LOS indicates length of stay, GSWs gunshot wounds, and SNOM selective nonoperative management

Among 7155 citations identified by the search, 41 studies [*n* = 22,847 patients with abdominal GSWs, of whom 6777 (29.7%) underwent SNOM] were included in the systematic review (Fig. 1). There was excellent inter-investigator agreement on inclusion of full-text articles (*κ*-statistic = 0.82; 95% CI = 0.45–1.00).

We found no controlled studies of SNOM that included a comparable control group of patients without diffuse peritonitis or hemodynamic instability that underwent mandatory laparotomy. Instead, all included studies examined outcomes of SNOM in a cohort of patients with abdominal GSWs to the anterior or other regions of the abdomen. Six studies reported outcomes of SNOM in patients with renal GSWs [30–34] while 5 reported SNOM outcomes in patients with hepatic GSWs [35–39]. Another three studies included patients with GSWs to the RUQ [40–42], one with GSWs to the back [43], and one with GSWs to the flank [44].

### Characteristics of the included studies/patients and SNOM practices

Characteristics of the included studies/patients and SNOM practices are shown in Table 1. Studies were published between 1966 and 2017, with the majority (63.4%) being published after the year 2000. Most were conducted at high-volume, level 1 trauma centers in the USA (70.7%) or South Africa (19.5%). Twenty-two (53.7%) studies were prospective and 19 (46.3%) were retrospective. Mean ages of patients ranged between 22.8 and 30.6 years, and 18 (43.9%) studies reported the mean ISS score, which ranged between 3 and 22.5.

**Table 1:** Characteristics of the 41 studies included in the systematic review

Study (setting, year)	Temporality	Study period	Trauma center level of care	No. of abdo GSWs	No. abdo GSWs treated with SNOM	Mean age (years)	Mean ISS	Reported SNOM characteristics			
								Frequency of physical exam	Serial examination done by	Serial laboratory examination	Use of CT
Reed et al. [65] (USA, 2017)	Retrospective	2003–2014	NR	127	63	26	17	NR	NR	NR	Selective
Peponis et al. [35] (USA, 2017)	Retrospective	1996–2015	I and II	922	215	NR	NR	Serial	Trauma team	NR	Selective
Starling et al. [59] (Brazil, 2015)	Prospective	2005–2014	NR	169	28	27.7	10.9	NR	NR	Yes	Mandatory
Navsaria et al. [45] (SA, 2015)	Prospective	2004–2009	I	1106	272	27.9	NR	Serial	NR	Yes	Selective
Laing et al. [30] (SA, 2014)	Retrospective	2012–2013	NR	80	15	28	NR	4-hourly for 12–24 h	Surgical trainee	NR	Mandatory
Cesar et al. [61] (Brazil, 2013)	Prospective	2005–2012	NR	NR	37	24	16	NR	NR	NR	Mandatory
Inaba et al. [46] (USA, 2012)	Prospective	2009–2011	NR	270	91	26.3	7.9	Serial	Trauma surgeon	Yes	Mandatory
Starling et al. [40] (Brazil, 2012)	Prospective	2005–2011	NR	NR	115	25.8	14.8	Short time intervals	NR	Yes	Mandatory
Zafar et al. [62] (USA, 2012)	Retrospective	2003–2008	I and II	12,707	3564	30	NR	NR	NR	NR	Selective
Hope et al. [63] (USA, 2012)	Retrospective	2006–2008	II	39	6	NR	NR	NR	NR	NR	Mandatory
Mnguni et al. [36] (SA, 2012)	Prospective	1998–2204	I	240	13	28.6	12.2	Serial	NR	NR	Selective
Schnüriger et al. [66] (USA, 2011)	Retrospective	2005–2007	I	125	11	28.1	22.5	NR	NR	NR	Mandatory
Fikry et al. [31] (USA, 2011)	Retrospective	1999–2009	I	125	38	25	14	NR	NR	NR	Selective
Bjurlin et al. [32] (USA, 2011)	Retrospective	2003–2008	NR	79	25	NR	NR	NR	NR	NR	Selective
Navsaria and Nicol [33] (SA, 2009)	Prospective	2004–2008	I	95	33	22.8	10.5	Serial for 48 h	NR	4-hourly hemoglobin level	Mandatory
Voelzke and McAninch [37] (USA, 2009)	Retrospective	1978–2008	I	201	51	27.8	NR	NR	NR	NR	Selective
Navsaria et al. [47] (SA, 2009)	Prospective	2004–2008	I	195	63	27.2	19.6	Serial for 48 h	NR	4-hourly hemoglobin level	Mandatory
Schmelzer et al. [64] (USA, 2008)	Prospective	NR	I	65	19	NR	NR	NR	NR	NR	Selective
Chamisa [48] (SA, 2008)	Prospective	Jan–Jun, 2008	NR	78	19	NR	NR	2-hourly interval	NR	NR	Selective
DuBose et al. [60] (USA, 2007)	Retrospective	1999–2005	I	644	144	28	15	NR	NR	Yes	Mandatory
MacLeod et al. [44] (USA, 2007)	Retrospective	1995–2003	I	896	396	27.8	16	Serial	NR	Yes	Selective
Demetriades et al. [38] (USA, 2006)	Prospective	2004–2006	I	107	39	NR	NR	Serial for 24–28 h	NR	Yes	Selective
Velmahos et al. [12] (USA, 2005)	Prospective	2002–2004	I	273	103	27	11	NR	NR	NR	Mandatory
Omoshoro-Jones et al. [49] (SA, 2005)	Prospective	2000–2002	I	124	33	25	NR	Serial for 48 h	NR	Yes	Mandatory
Múnera et al. [11] (USA, 2004)	Prospective	2000–2002	I	47	36	23.5	NR	NR	NR	NR	Mandatory
Velmahos et al. [39] (USA, 2001)	Retrospective	1993–2000	I	1856	792	25	3	Frequent for 12–24 h	Same resident with active involvement of attending staff	NR	Selective
Demetriades et al. [50] (USA, 1999)	Retrospective	1994–1998	I	52	16	30	17.5	Serial for 24 h	NR	Yes	Mandatory
Velmahos et al. [67] (USA, 1998)	Retrospective	1994–1995	I	54	4	26.2	20	NR	Trauma team	NR	Mandatory
Adesanya et al. [57] (Nigeria, 1998)	Prospective	1992–1996	NR	78	14	30	NR	NR	Consultant surgeon	NR	NR
Velmahos et al. [43] (USA, 1997)	Prospective	1994–1995	I	192	130	24	NR	Serial	NR	Yes	Selective
Demetriades et al. [51] (USA, 1997)	Prospective	1994–1995	I	309	106	24.7	NR	24 h	Admitting surgeon	Yes	Selective
Wessells et al. [34] (USA, 1997)	Retrospective	1980–1995	NR	45	4	30.6	NR	NR	NR	Yes	Selective
Chmielewski et al. [41] (USA, 1995)	Prospective	1991–1994	I	184	12	NR	NR	48 h	NR	Yes	Selective
Renz and Feliciano [42] (USA, 1994)	Prospective	1990–1993	I	32	13	NR	NR	Frequent	Resident, at least twice a day by the primary investigator	Yes	Selective
Demetriades et al. [52] (USA, 1991)	Prospective	1988–1990	NR	146	41	28	NR	Regular	Same surgeon	NR	NR
Muckart et al. [53] (SA, 1990)	Prospective	Jul–Aug, 1988	NR	111	22	24	NR	2-hourly	NR	NR	NR
McAlvanah and Shaftan [54] (USA, 1978)	Prospective	1963–1971	NR	221	101	NR	NR	Every ½ to 1 h for 24–36 h	NR	NR	NR
Lowe et al. [55] (USA, 1977)	Retrospective	1972–1974	NR	362	55	27.8	NR	48 h	Surgical resident supervised by attending staff	NR	Selective
Taylor [68] (USA, 1973)	Retrospective	1962–1970	NR	254	8	NR	NR	NR	NR	NR	NR
Richter and Zaki [58] (USA, 1967)	Retrospective	1957–1966	NR	35	13	NR	NR	NR	Surgeon	NR	NR
Ryzoff et al. [56] USA, 1966)	Retrospective	1956–1963	NR	50	17	NR	NR	Frequent	NR	NR	NR

*abdo* abdominal, *CT* computed tomography, *GSW* gunshot wound, *ISS* Injury Severity Score, *NR* not reported, *SA* South Africa, *SNOM* selective nonoperative management, *USA* United States of America

In all studies, hemodynamically stable patients without a reduced level of consciousness and no signs of peritonitis were selected for SNOM. Twenty-one (51.2%) studies reported that patients were admitted to a dedicated, monitored observation area for 12–48 h before being transferred to a floor bed or discharged from hospital [30, 33, 38–56]. Four (19%) of these 21 studies reported that patients underwent serial physical examinations at time intervals ranging from every half-hour to 4 h [30, 48, 53, 54]. Physical examinations were reportedly performed by an attending trauma surgeon in 5 (12.2%) studies [46, 51, 52, 57, 58] and a surgical resident in 4 (9.8%) studies [30, 39, 42, 55]. Sixteen (39.0%) studies reported that patients also had serial measurements of hemoglobin, hematocrit, and white blood cell (WBC) counts during the post-GSW observation period [33, 34, 38, 40–47, 49–51, 59, 60]. Patients selected for SNOM were evaluated with abdominopelvic CT scans with intravenous contrast (in either a mandatory or selective fashion) in 33 (80.5%) studies [11, 12, 30–34, 36–41, 43–52, 55, 59–67].

### Risk of bias assessment

Table 2 summarizes the risk of bias assessment for all included studies. Most studies had a low to moderate risk of bias. Six (14.6%) studies demonstrated a high risk of bias in at least 1 modified QUIPS tool domain [12, 55, 56, 58, 62, 68]. Thirty-four (82.9%) studies showed a moderate-to-high risk of study attrition bias due to inadequate reporting of information about whether patients were lost to follow-up [12, 30–34, 36–41, 43–48, 50–64, 66–68]. Twenty (48.7%) studies demonstrated a moderate-to-high risk of prognostic factors measurement bias because (1) the authors did not report data on the grade of injury suffered by study patients in 18 (43.9%) studies [11, 12, 30, 39, 41–45, 48, 51–55, 57, 62, 68], ISS score of the patients in 15 (36.6%) studies [11, 38, 40–43, 48, 49, 51–55, 58, 68], or age of the patients in 4 (9.8%) studies [41, 48, 53, 68]; (2) there was no standardized policy for SNOM adopted in 9 (22.0%) studies [12, 31, 32, 43, 54, 58, 62–64]; or (3) patients were managed operatively based on the decision of the attending surgeon rather than a defined protocol or decision algorithm in 3 (7.3%) studies [31, 58, 63]. Thirteen (31.7%) studies showed a moderate-to-high risk of outcome measurement bias because (1) the definition of failure of SNOM was based on the timing from admission to the operating room in 2 (4.9%) studies [31, 62]; (2) failure of SNOM was solely based on CT findings without considering physical examination findings in 3 (7.3%) studies [12, 32, 55]; or (3) a clear definition of failure of SNOM was not provided in 8 (19.5%) studies [34, 37, 38, 56, 58, 63, 64, 68].

**Table 2:** Risk of bias assessment of the 41 included studies

Study	Study participation	Study attrition	Prognostic factors measurement	Outcome measurement	Statistical analysis and reporting
Reed et al. [65]	Low	Moderate	Low	Low	Low
Peponis et al. [35]	Moderate	Moderate	Low	Low	Low
Starling et al. [59]	Low	Moderate	Low	Low	Low
Navsaria et al. [45]	Low	Moderate	Moderate	Low	Low
Laing et al. [30]	Moderate	Moderate	Low	Low	Moderate
Cesar et al. [61]	Low	Moderate	Low	Low	Low
Inaba et al. [46]	Low	Moderate	Low	Low	Low
Starling et al. [40]	Moderate	Moderate	Low	Low	Low
Zafar et al. [62]	Low	High	Moderate	Moderate	Low
Hope et al. [63]	Low	Moderate	Moderate	Moderate	Low
Mnguni et al. [36]	Moderate	Moderate	Low	Low	Low
Schnüriger et al. [66]	Low	Moderate	Low	Low	Moderate
Fikry et al. [31]	Low	Moderate	Moderate	Moderate	Low
Bjurlin et al. [32]	Moderate	Moderate	Moderate	Moderate	Moderate
Navsaria and Nicol [33]	Low	Moderate	Low	Low	Low
Voelzke and McAninch [37]	Low	Moderate	Low	Moderate	Moderate
Navsaria et al. [47]	Low	Moderate	Low	Low	Low
Schmelzer et al. [64]	Low	Moderate	Moderate	Moderate	Low
Chamisa [48]	Low	Moderate	Moderate	Low	Low
DuBose et al. [60]	Low	Moderate	Low	Low	Low
MacLeod et al. [44]	Low	Moderate	Low	Low	High
Demetriades et al. [38]	Moderate	Moderate	Moderate	Moderate	Low
Velmahos et al. [12]	Low	Moderate	Moderate	Moderate	Moderate
Omoshoro-Jones et al. [49]	Low	Low	Low	Low	Low
Múnera et al. [11]	Low	Low	Low	Low	Moderate
Velmahos et al. [39]	Low	Moderate	Low	Low	Low
Demetriades et al. [50]	Low	Moderate	Low	Low	Moderate
Velmahos et al. [67]	Low	Moderate	Low	Low	Low
Adesanya et al. [57]	Low	Moderate	Moderate	Low	Moderate
Velmahos et al. [43]	Low	Moderate	Moderate	Low	Low
Demetriades et al. [51]	Low	Moderate	Low	Low	Moderate
Wessells et al. [34]	Low	Moderate	Moderate	Moderate	Moderate
Chmielewski et al. [41]	Moderate	Moderate	Moderate	Low	Moderate
Renz and Feliciano [42]	Low	Low	Moderate	Low	Low
Demetriades et al. [52]	Low	Moderate	Moderate	Low	Moderate
Muckart et al. [53]	Low	Low	Low	Low	Moderate
McAlvanah and Shaftan [54]	Low	Moderate	Moderate	Low	Low
Lowe et al. [55]	Moderate	Moderate	Moderate	High	High
Taylor [68]	Moderate	High	High	High	High
Richter and Zaki [58]	Moderate	Moderate	High	Moderate	Moderate
Ryzoff et al. [56]	Moderate	Moderate	Moderate	Moderate	Moderate

One study included 12,707 patients that sustained abdominal GSWs between 2002 and 2008 and were included in the National Trauma Data Bank (NTDB) [62], which contains data collected from approximately 900 trauma centers in the USA. This study likely included duplicate patients from other American studies published during the same period [9, 12, 31, 32, 37, 44, 60, 63, 66]. Further, a number of other studies used the same data source to identify patients, but looked at different outcomes and/or patient populations [33, 39, 40, 43, 45, 50, 59, 67]. However, it was not possible to determine the amount of overlap or duplication of patients with certainty between these studies, and therefore, the influence of overlap was explored in a post hoc sensitivity analysis described below.

### Outcomes associated with use of SNOM for abdominal GSWs

#### Primary outcome

**Fig. 2:**
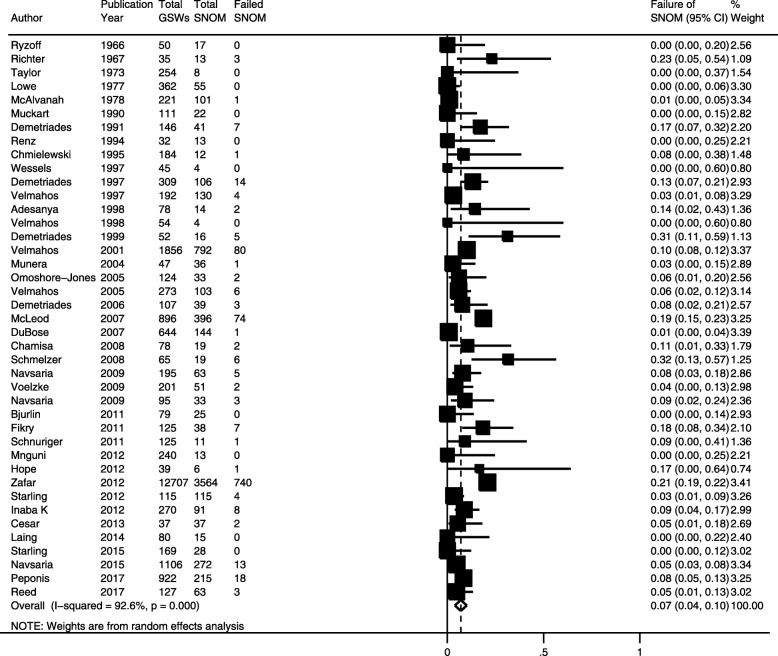
Pooled risk of failure in civilians undergoing selective nonoperative management of abdominal gunshot wounds

The pooled estimate of the risk of failure of SNOM for abdominal GSWs across 28 studies that reported data on this outcome was 7.0% (95% CI = 3.9–10.1%) (Fig. 2). There was a high degree of heterogeneity between these studies (*I*^2^ = 92.6%, homogeneity *p* < 0.001). In patients who failed SNOM, the pooled estimate of the risk of therapeutic laparotomy was 68.0% (95% CI = 58.3–77.7%; *I*^2^ = 91.5%; homogeneity *p* < 0.001) while that of unnecessary laparotomy was 28.1% (95% CI = 19.0–37.1%; *I*^2^ = 90.6%, homogeneity *p* < 0.001).

Twenty studies (48.8%) reported the length of time from admission to delayed laparotomy in patients that failed SNOM. The weighted mean and median time between admission and delayed laparotomy in patients selected for a trial of SNOM (*n* = 16 studies, 39.0%) was 40.6 and 21.2 h, respectively [12, 31, 35, 38, 40, 41, 43, 45–47, 49, 51, 60, 63, 65, 66].

Indications for delayed laparotomy (as well as intraoperative findings) in patients who failed SNOM were described in 17 (41.5%) of the included studies and are described in detail in the table in Additional file 3: digital content S3. These most commonly included development of peritonitis (38.8%) [12, 31, 33, 35, 46–48, 50, 52], worsening abdominal tenderness (35.7%) [35, 41, 43, 45, 51], or fever (11.2%) [33, 41, 43, 45, 46, 48, 63]. Less common reasons included development of new tachycardia (8.2%) [41, 46, 47] or hypotension (1.0%) [60]. Others included rising WBC counts (6.1%) [46, 63] or falling hematocrit levels (6.1%) [31–33, 37, 46, 47, 50, 51].

#### Secondary outcomes

##### Morbidity and mortality of patients that underwent SNOM

Pooled estimates of the risk of complications associated with SNOM are reported in Table 3. Of these, atelectasis and GSW infections were most common, with pooled risk estimates of 21.2% (95% CI = 7.0–35.4%, *I*^2^ = 0.0%, homogeneity *p* = 0.36) and 6.0% (95% CI = 3.1–8.9%, *I*^2^ = 0%, homogeneity *p* = 0.71), respectively. The pooled estimate of the risk of any intrathoracic complication (pneumothorax, hemothorax, empyema, or pleural effusion) was 11.6% (95% CI = 3.5–19.7%; *I*^2^ = 79.0%; homogeneity *p* = 0.005). Three studies (7.7%) reported biliary fistula formation after SNOM in patients with liver GSWs, with a pooled risk of 3.5% (95% CI = 1.0–8.0%; *I*^2^ = 61.8%; homogeneity *p* = 0.07) [12, 33, 42].

**Table 3:** Pooled risk of complications associated with selective nonoperative management of abdominal gunshot wounds

Complications	No. of studies	No. of patients	Pooled risk, % (95% CI)	*I*^2^ statistic, %	*p* value for the test of homogeneity
Pneumonia	7	20	1.4 (0.1–2.7)	61.7	0.02
Atelectasis	2	7	21.2 (7.0–35.4)	0	0.36
ARDS	5	8	1.0 (0–2.6)	43.8	0.13
Sepsis	4	6	0.3 (0–1.0)	36.2	0.20
Any intra-thoracic complication*	5	25	11.6 (3.5–19.7)	79.0	0.001
Any intra-abdominal collection^†^	7	16	0.8 (0.3–1.2)	0	0.49
Hematuria	2	3	1.5 (0.5–2.5)	98.8	0.003
Biliary fistula	3	8	3.5 (0–8.0)	99.5	< 0.001
Gunshot wound infection	2	3	6.0 (3.1–8.9)	0	0.71
Deep venous thrombosis	2	2	0.5 (0–1.4)	0	0.40
Ileus	4	4	0.2 (0–0.4)	0	0.43
Abdominal compartment syndrome	1	1	6.0 (0.2–30)	NA	NA
Necrotizing fasciitis	1	1	3.0 (0.1–16.0)	NA	NA

*ARDS* acute respiratory distress syndrome

*Intra-thoracic complications included pneumothorax, hemothorax, empyema, and pleural effusion(s)

^†^Intra-abdominal collection included abscess, hematoma, urinoma, or biloma

**Fig. 3:**
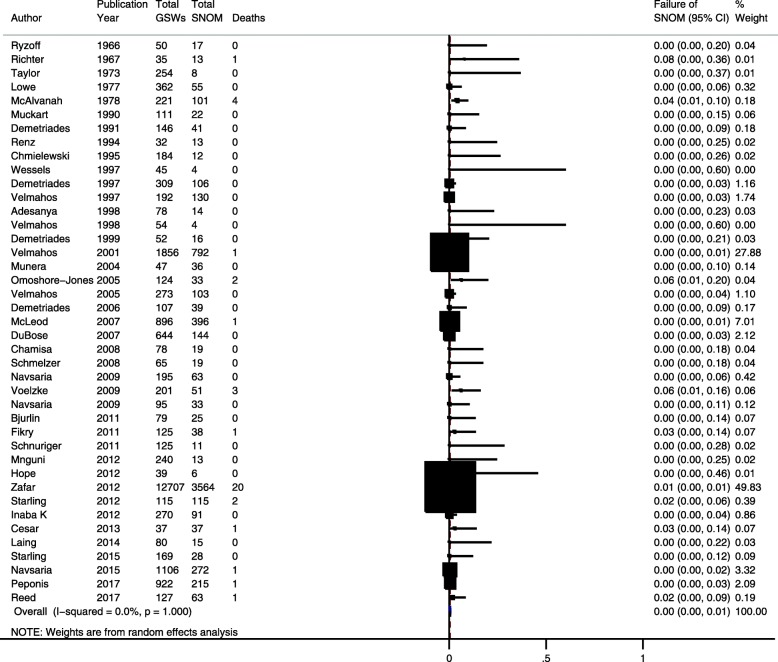
Pooled risk of mortality in civilians undergoing selective nonoperative management of abdominal gunshot wounds

Among 10 (24.4%) studies that reported data on in-hospital mortality [31, 35, 39, 40, 44, 45, 54, 58, 62, 65], the pooled risk of in-hospital death after SNOM for abdominal GSWs was 0.4% (95% CI = 0.2–0.6%). There was no evidence of heterogeneity in this estimate (*I*^2^ = 0%; homogeneity *p* > 0.99) (Fig. 3).

##### Hospital length of stay

Twenty-nine studies (70.7%) reported the mean hospital LOS among patients who underwent SNOM, which varied from 2 to 20 days. The weighted average hospital LOS was 5.9 days. However, in patients with isolated abdominal GSWs without any associated extra-abdominal injuries, the weighted median hospital LOS was 2.3 days. Conversely, the weighted median hospital LOS in patients who failed SNOM or developed complications was 10.1 days.

### Subgroup and sensitivity analyses and meta-regression

Table 4 details results of stratified meta-analyses and meta-regression of variables associated with failure of SNOM for abdominal GSWs. There was no difference in the pooled risk of SNOM failure between prospective and retrospective studies or those conducted in the USA, South Africa, or other countries. Risks of failure of SNOM were lowest in studies that evaluated patients with right thoracoabdomen (3.4%; 95% CI = 0–7.0%; *I*^2^ = 0%; homogeneity *p* = 0.45), flank (7.0%; 95% CI = 3.9–10.1%), and back (3.1%; 95% CI = 0–6.5%) GSWs and highest in those that evaluated patients with anterior abdomen (13.2%; 95% CI = 6.3–20.1%) GSWs. Estimates of the pooled risk of failure in studies where serial physical examinations were reportedly done by attending trauma surgeons were approximately one-third that of those reported in studies where physical examinations were done by surgical residents. When studies were divided according to the policy of CT use, the pooled estimate of SNOM failure in studies of patients undergoing selective abdominopelvic CT was approximately double that of the pooled estimate of SNOM failure in studies of patients undergoing mandatory CT.

**Table 4:** Stratified meta-analyses and meta-regression of variables associated with failure of selective nonoperative management of abdominal gunshot wounds

Comparison		No. of studies	Pooled estimate of SNOM failure, % (95% CI)	*I*^2^ statistic, %	Meta-regression *p* value
Study temporality and setting	Prospective	22	4.3 (2.7–6.0)	24.4	0.27
	Retrospective	19	8.0 (2.8–13.3)	95.6	
	Conducted in the USA	29	8.1 (4.2–12.1)	94.3	NA
	Conducted in South Africa	8	4.7 (2.5–6.9)	0	0.32*
	Conducted in other countries	4	7.0 (3.9–10.1)	0	0.35*
Study patient injury patterns	Abdominal GSWs	24	7.3 (3.0–11.5)	95.1	NA
	Liver GSWs	5	6.6 (0.0–13.3)	49.8	0.99^†^
	Renal GSWs	6	3.5 (0–7.3)	0	0.39^†^
	Back GSWs	1	3.1 (0–6.5)	NA	0.52^†^
	Flank GSWs	1	7.0 (3.9–10.1)	NA	0.09^†^
	Anterior abdomen GSWs	1	13.2 (6.3–20.1)	NA	0.51^†^
	Right thoracoabdomen GSWs	3	3.4 (0–7.0)	0	0.45^†^
Reported SNOM practices	SNOM by attending surgeon	5	2.1 (7.8–16.3)	0	0.07
	SNOM by surgical resident	4	7.2 (3.9–10.5)	89.5	
	Mandatory use of CT	15	4.2 (1.9–6.5)	33.5	0.08
	Selective use of CT	19	8.3 (3.9–12.8)	94.4	

*CI* confidence interval, *CT* computed tomography, *GSW* gunshot wound, *SNOM* selective nonoperative management

*Compared to the estimate associated with USA

^†^Compared to abdominal GSWs

A sensitivity analysis excluding the results of studies that reported potentially overlapping patient outcome data (including those that may have overlapped with those recruited into the study that analyzed patients included in the NTDB) yielded a similar pooled estimate of the risk of failure of SNOM for abdominal GSWs (6.7%, 95% CI = 2.5–10.9%, *I*^2^ = 94.4%, homogeneity *p* < 0.001) to the pooled estimate that included data from all studies reporting data on this outcome.

### Publication bias

**Fig. 4:**
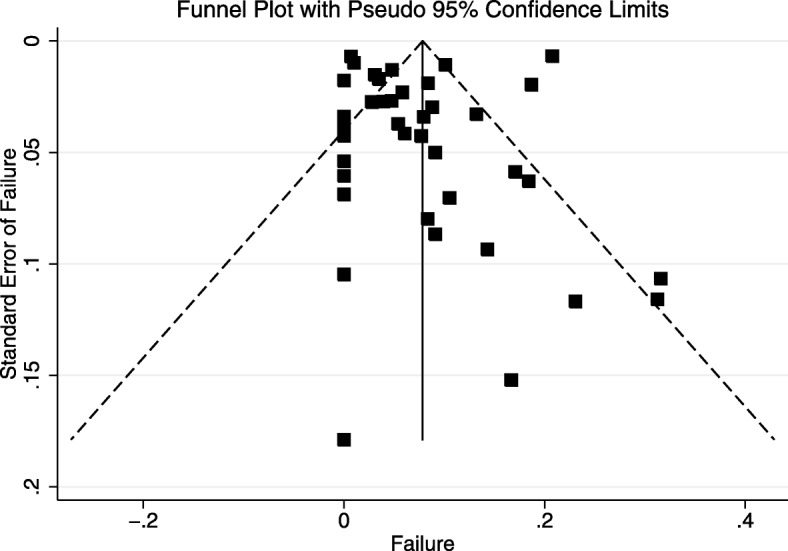
Funnel plot of the risk of failure of selective nonoperative management versus the associated standard error of the risk of failure

Inspection of the funnel plots of the reported risks of failure of SNOM versus the standard error across the included studies revealed that smaller studies may have reported higher risks of failure than larger studies (Fig. 4). Further, Begg’s test was significant (*p* = 0.008) for funnel plot asymmetry. However, using Trim and Fill methods, the publication bias-adjusted summary estimate of the risk of failure of SNOM was unchanged (7.2%; 95% CI = 4.2%–10.1%) from the unadjusted estimate (7.0%; 95% CI = 3.9–10.1%), providing evidence that publication bias likely had little influence on the pooled results.

## Discussion

This systematic review of 41 studies involving 22,847 civilians with abdominal GSWs is the first to comprehensively meta-analyze outcomes associated with use of SNOM in this patient population. Our findings suggest that highly experienced trauma centers have safely treated greater than 90% of the patients included in these studies nonoperatively. Importantly, in all studies, only hemodynamically stable patients without a reduced level of consciousness and no signs of peritonitis were selected for SNOM. SNOM may be more successful among patients with GSWs to the right thoracoabdomen, flank, or back than the anterior abdomen and when serial physical examinations are performed by attending trauma surgeons. This practice also appears to be more successful in patients who undergo mandatory abdominopelvic CT scans and in those with injuries proven to involve the kidney on imaging. Although SNOM has been linked with development of atelectasis, GSW infections, and biliary fistulae in patients with GSWs to the liver, these complications are uncommon and appear to be less frequent than that historically reported after mandatory laparotomy in similar patient populations [69–71].

As most of the included studies were conducted at high-volume, level 1, academic trauma centers in the USA, our results may not be generalizable to centers without the necessary structures and processes to manage these patients. Many of the centers conducting research on SNOM described dedicated, monitored areas to observe and examine patients with abdominal GSWs in the initial hours after injury (and therefore these systems may be required to assure timely rescue of patients who fail SNOM). Of those who failed SNOM, the most frequent reasons for delayed laparotomy included development of peritonitis or worsening abdominal tenderness, each of which was mostly commonly detected within the first 24–48 h of admission through close clinical monitoring. It is interesting that 28% of patients who failed SNOM across the included studies underwent unnecessary laparotomy, which suggests that success of SNOM may potentially benefit from studies focused on creating appropriate indications for delayed operation during SNOM [72].

Our findings identified that mandatory use of abdominopelvic CT was associated with a failure rate of SNOM that was approximately half of that reported for selective use of CT scanning. Further, the number of unnecessary laparotomies in patients who failed SNOM was higher in studies that used a selective policy of CT scanning. These findings suggest that use of CT may confer a higher degree of confidence to surgeons managing patients with an equivocal clinical exam or concerning bullet trajectory. Whereas CT may be argued to be of minimal help to patients with anterior abdominal GSWs who are clearly hemodynamically stable and have no abdominal tenderness, there are situations in which CT will provide much-needed, additional information to guide surgical decision-making. These include patients with suspected tangential abdominal wounds, back or flank GSWs who may have retroperitoneal injuries, or RUQ GSWs who may have isolated hepatic trauma. These findings may support the level 2 recommendation made by the Eastern Association for the Surgery of Trauma in their 2010 guideline that abdominopelvic CT be strongly considered in patients undergoing SNOM of penetrating abdominal wounds (especially those with GSWs to the flank, back, and RUQ) [13].

Common complications of SNOM, as reported across the included studies, were frequently relatively minor and may be divided into those that are thoracic (pneumothorax, hemothorax, or pleural effusion) and abdominal (GSW infection and biliary fistulae in patients with GSWs to the liver). As the risk of complications in patients undergoing mandatory laparotomy has been reported to be as high as 22–41% [4, 5], the frequency of complications in patients undergoing SNOM in experienced trauma centers (13% across 11 studies) may be similar or even lower than mandatory laparotomy. However, when patients develop complications after SNOM for abdominal GSWs, their mean hospital LOS appears to increase from approximately 2 (in patients without associated extra-abdominal injuries) or 6 (in patients with associated extra-abdominal injuries) to 10 days. In particular, SNOM of liver GSWs was associated with a risk of biliary fistulae (6.3%) and pulmonary complications (15.9%).

This systematic review has several limitations. First, we identified no controlled studies comparing outcomes of conducting SNOM versus mandatory laparotomy in hemodynamically stable patients with abdominal GSWs without diffuse peritonitis. Second, although we were unable to determine the amount of duplication of included patient outcome data with certainty, a sensitivity analysis excluding the results of studies that reported potentially overlapping data yielded a similar pooled estimate of the failure of SNOM. Third, few studies examined associations between the use of angioembolization/endovascular interventions and SNOM failure or provided details of whether reported complications were directly attributable to a failure of SNOM. Fourth, some studies did not report their methods of conducting SNOM. Thus, the management of each case was ultimately dictated by attending surgeons, which likely introduced unmeasured variability in practice and outcomes. Finally, some studies included patients in the SNOM group that only had tangential and superficial abdominal GSWs without peritoneal breach, which likely improved the reported outcomes of SNOM.

## Conclusion

In conclusion, this systematic review and meta-analysis of 41 cohort studies involving 22,847 patients with abdominal GSWs (of whom 6777 patients underwent SNOM) suggests that SNOM is safe when conducted in experienced trauma centers. The practice may be especially useful when serial physical examinations are performed by attending trauma surgeons and in patients with GSWs involving the right thoracoabdomen, flank, or back instead of the anterior abdomen and those proven to involve the kidney on imaging. The most frequent reasons for delayed laparotomy in patients undergoing SNOM include development of peritonitis or worsening abdominal tenderness, each of which is likely to be detected within the first 24–48 h of admission across hospitals with the necessary experience and/or resources to ensure timely rescue of patients who fail SNOM. Mandatory use of abdominopelvic CT may increase the success of SNOM, potentially by increasing clinician confidence when managing patients with an equivocal clinical examination or identifying/characterizing retroperitoneal/isolated hepatic injuries in those with GSWs to the flank, back, or RUQ.

## Additional files

Additional file 1: Digital content S1. Completed Meta-Analysis of Observational Studies in Epidemiology (MOOSE) checklist. (DOCX 16 kb)

Additional file 2: Digital content S2. Literature search strategies. (DOCX 15 kb)

Additional file 3: Digital content S3. Indications for delayed laparotomy in patients undergoing selective nonoperative management and findings at the time of operation. (DOCX 29 kb)

## Abbreviations

CI: Confidence interval
CT: Computed tomography
GSW: Gunshot wound
ISS: Injury Severity Scale
LOS: Length of stay
MeSH: Medical Subject Heading
MOOSE: Meta-analysis of Observational Studies in Epidemiology
QUIPS: Quality in Prognosis Studies
RUQ: Right upper quadrant
SNOM: Selective nonoperative management
WBC: White blood cell
