# Supplementary material for: Outcomes of selective nonoperative management of civilian abdominal gunshot wounds: a systematic review and meta-analysis
Source: World J Emerg Surg. 2018 Nov 27;13:55. doi: 10.1186/s13017-018-0215-0 (PMC6260713; doi:10.1186/s13017-018-0215-0)
Supplement: Supplementary file 1 — Digital content S1. Completed Meta-Analysis of Observational Studies in Epidemiology (MOOSE) checklist. (DOCX 16 kb) [file 13017_2018_215_MOESM1_ESM.docx]

**Additional file 1: Digital Content S1. Completed Meta-analysis of Observational Studies in Epidemiology (MOOSE) Checklist.**

Al Rawahi AN, Al Hinai FA, Boyd JM, Doig CJ, Ball CG, Velmahos G, Kirkpatrick AW, Navsaria PH, Roberts DJ. Outcomes of Selective Nonoperative Management of Civilian Abdominal Gunshot Wounds: A Systematic Review and Meta-Analysis.

| **Item** | **Page** |
| --- | --- |
| Reporting of background should include |  |
| Problem definition | 6-7 |
| Hypothesis statement | 7 |
| Description of study outcomes | 10 |
| Type of exposure or intervention used | 8-9 |
| Type of study designs used | 8-9 |
| Study population | 8-9 |
| Reporting of study search strategy should include |  |
| Qualifications of searchers (e.g., librarians and investigators) | 8 |
| Search strategy, including time period included in the synthesis and keywords | 8 and SDC 2 |
| Efforts to include all available studies, including contact with authors | 8 |
| Databases and registries searched | 8 |
| Search software used, name and version, including special features used | 8 |
| Use of hand searching (e.g., reference lists of obtained articles) | 8 |
| List of citations located and those excluded, including justification | Figure 1 |
| Method of addressing articles published in languages other than English, including justification | 8 |
| Method of handling abstracts and unpublished studies | 9 |
| Description of any contact with authors | 8 |
| Reporting of methods should include |  |
| Description of relevance of appropriateness of studies assembled for assessing the hypothesis to be tested | 8-9 |
| Rationale for the selection and coding of data (e.g., sound clinical principles and convenience) | 9-10 |
| Documentation of how data was classified and coded (e.g., multiple raters, blinding, and interrater reliability) | 9-10 |
| Assessment of confounding (e.g., comparability of cases and controls in studies where appropriate) | Not applicable |
| Assessment of study quality | 10-11 and SDC 3 |
| Assessment of heterogeneity | 11-12 |
| Description of statistical methods | 11-12 |
| Reporting of results should include |  |
| Graphic summarizing individual study estimates and overall estimate | Figure 2 and Figure 3 |
| Table giving descriptive information for each study included | Table 1 |
| Results of sensitivity testing (e.g., subgroup analyses) | 17-18 and Table 4 |
| Indication of statistical uncertainty of findings | Presented with confidence intervals |
| Reporting of discussion should include |  |
| Quantitative assessment of bias (e.g., publication bias) | 18 and SDC 5 |
| Justification of exclusion (e.g., exclusion of non-English language citations) | Figure 1 |
| Assessment of quality of included studies | 14-15 and Table 2 |
| Reporting of conclusions should include |  |
| Consideration of alternative explanations for observed results | 19-22 |
| Generalization of the conclusions (i.e., appropriate for the data presented and within the domain of the literature review) | 19-22 |
| Guidelines for future research | 19-22 |
| Disclosure of funding source | Not applicable |

Where SDC indicates Supplemental Digital Content.
