# Supplementary material for: Outcomes of selective nonoperative management of civilian abdominal gunshot wounds: a systematic review and meta-analysis
Source: World J Emerg Surg. 2018 Nov 27;13:55. doi: 10.1186/s13017-018-0215-0 (PMC6260713; doi:10.1186/s13017-018-0215-0)
Supplement: Supplementary file 2 — Digital content S2. Literature search strategies. (DOCX 15 kb) [file 13017_2018_215_MOESM2_ESM.docx]

**|Additional file 2: Digital Content S2. Search Strategy**

*PUBMED:*

1.conservative OR non operative OR nonoperative OR non-operative 150013

2.trauma OR traumas OR traumatic 1061910

3.penetratine OR penetrates OR penetrate 18522

4.gunshot OR shotgun OR shot OR high kinetic OR high speed injuries 164093

5.wound OR wounds OR wounded OR injury OR injuries 1129342

6.2 OR 3 OR 4 OR 5

7.Spleen OR splenic OR liver OR hepatic OR kidney OR renal/ 1979047

8.Nonoperative OR non operative OR non surgical OR nonsurgical OR conservative

9.Abdominal injuries or abdominal injury OR abdomen / 175681

10. 7 OR 9

11. 6 AND 8 AND 10

*MEDLINE*

1.conservative OR non operative OR nonoperative OR non-operative

2.trauma OR traumas OR traumatic

3.penetratine OR penetrates OR penetrate 15820

4.gunshot OR shotgun OR shot OR high kinetic OR high speed injuries

5.wound OR wounds OR wounded OR injury OR injuries 1129432

6.2 OR 3 OR 4 OR 55564

7.Spleen OR splenic OR liver OR hepatic OR kidney OR renal/ 2213423

8.Nonoperative OR non operative OR non surgical OR nonsurgical OR conservative 7654

9.Abdominal injuries or abdominal injury OR abdomen / 92230

10. 7 OR 9

11. 6 AND 8 AND 10

*EMBASE:*

1.conservative OR non operative OR nonoperative OR non-operative OR non surgical 8404

2.trauma OR traumas OR traumatic 22078

3.penetratine OR penetrates OR penetrate 160553

4.gunshot OR shotgun OR shot OR high kinetic OR high speed injuries 10022

5.wound OR wounds OR wounded OR injury OR injuries 75862

6.2 OR 3 OR 4 OR 43356

7.Spleen OR splenic OR liver OR hepatic OR kidney OR renal/ 14634

8.Abdominal injuries or abdominal injury OR abdomen / 15066

9. 7 OR 8

10. 6 AND 8 AND 9

*COCHRANE LIBRARY*

1.Controlled Clinical Trial/ or Clinical Trial/ or Randomized Controlled Trial 212434

2.conservative OR non operative OR non-operative  5744

3.penetrating or penetrate OR penetrates 376

4.gunshot or shotgun or shot or high kinetic or high speed injuries 886

5. ( 2 OR 3 OR 4) 2562

6. (1 & 5 )
