# Supplementary material for: Outcomes of selective nonoperative management of civilian abdominal gunshot wounds: a systematic review and meta-analysis
Source: World J Emerg Surg. 2018 Nov 27;13:55. doi: 10.1186/s13017-018-0215-0 (PMC6260713; doi:10.1186/s13017-018-0215-0)
Supplement: Supplementary file 3 — Digital content S3. Indications for delayed laparotomy in patients undergoing selective nonoperative management and findings at the time of operation. (DOCX 29 kb) [file 13017_2018_215_MOESM3_ESM.docx]

**Additional file 3: Digital Content S3. Indications for Delayed Laparotomy.**

| **Study** | **No. Patients treated with SNOM** | **No. Delayed Laparotomy** | **No. Therapeutic laparotomy** | **Time to Delayed Laparotomy** | **Reasons for Failure of SNOM** | **Laparotomy Findings** |
| --- | --- | --- | --- | --- | --- | --- |
| Reed et al. ^66^ | 63 | 3 | 3 | Patient (1) Day 44  Patient (2) Day 2  Patient (3) Day 3 | NR | Patient (1) liver abscess debridement  Patient (2) bile peritonitis  Patient (3) Day 3 left diaphragm injury |
| Peponis et al. ^35^ | 215 | 18 | 17 | 141 minutes to 48 hours. Mean 12.5 hours | Patient (1) peritonitis  Patient (2) increased abdominal pain  Patient (3) CT showed liver injury and possible diaphragm injury  Patient (4) CT showed liver injury and possible diaphragm injury  Patient (5) CT showed liver injury and diaphragm injury  Patient (6) persistent nausea & vomiting  Patient (7) CT showed diaphragm injury and suspicion of colon injury  Patient (8) CT showed splenic and diaphragmatic  Patient (9) peritonitis  Patient (10) CT suspicious of stomach injury  Patient (11) CT suspicious of stomach and pancreatic injury  Patient (12) increased abdominal pain  Patient (13) peritonitis  Patient (14) abdominal pain and tachycardia  Patient (15) CT showed left diaphragm and possible stomach injury  Patient (16) peritonitis  Patient (17) CT suspicious of colon injury  Patient (18) peritonitis | Patient (1) small bowel injury  Patient (2) stomach and diaphragm injury  Patient (3) liver and diaphragm injury  Patient (4) liver and diaphragm injury  Patient (5) liver and diaphragm injury  Patient (6) small bowel injury  Patient (7) diaphragm and transverse colon injury  Patient (8) diaphragm injury, splenectomy  Patient (9) small bowel injury  Patient (10) stomach injury  Patient (11) stomach perforation and pancreatic contusion  Patient (12) none therapeutic  Patient (13) small and large bowel injury  Patient (14) cecal perforation  Patient (15) stomach perforation and diaphragm injury  Patient (16) small bowel injury  Patient (17) colon injury  Patient (18) small bowel injury |
| Starling et al.^64^ | 28 | 0 | 0 | NA | NA | NA |
| Navsaria et al. ^50^ | 272 | 13 | 10 | 4 hours to 11 days.  Mean 30 hours | Patient (1) peritonitis  Patient (2) peritonitis  Patient (3) peritonitis  Patient (4) peritonitis  Patient (5) peritonitis, increased blood transfusion  Patient (6) respiratory failure  Patient (7) peritonitis  Patient (8) peritonitis  Patient (9) respiratory failure  Patient (10) peritonitis  Patient (11) peritonitis  Patient (12) peritonitis  Patient (13) peritonitis | Patient (1) negative laparotomy  Patient (2) splenectomy and diaphragm repair  Patient (3) small bowel injury  Patient (4) negative laparotomy  Patient (5) distal pancreatectomy, splenectomy, liver drained  Patient (6) liver drained  Patient (7) diaphragm injury, liver drained  Patient (8) colon injury, liver drained  Patient (9) nephrectomy  Patient (10) splenectomy, nephrectomy  Patient (11) nephrectomy  Patient (12) small bowel injury  Patient (13) colon injury, diaphragm injury, pancreas drained |
| Laing et al.^30^ | 15 | 0 | 0 | NA | NA | NA |
| Cesar et al.^45^ | 37 | 2 | 2 | NR | NR | Liver injury with choleperitoneum (2) |
| Inaba et al.^51^ | 91 | 8 | 6 | Patient (1) 1:52 hours  Patient (2) 4:19 hours  Patient (3) 21:55 hours  Patient (4) 5:14 hours  Patient (5) 2:32 hours  Patient (6) 23:21 hours  Patient (7) 11:31 hours  Patient (8) 6:41 hours | Patient (1) tachycardia and peritonitis  Patient (2) fever, tachycardia and peritonitis  Patient (3) tachycardia, peritonitis and increased WBC  Patient (4) tachycardia, peritonitis and increased WBC  Patient (5) tachycardia and peritonitis  Patient (6) fever, tachycardia, peritonitis and increased WBC  Patient (7) fever and increased WBC  Patient (8) increased WBC | Patient (1) mesocolon hematoma  Patient (2) liver and stomach injury  Patient (3) colon injury  Patient (4) liver, stomach and colon injury  Patient (5) liver and stomach injury  Patient (6) liver injury  Patient (7) rectal injury  Patient (8) liver and stomach injury |
| Starling et al.^40^ | 115 | 4 | 3 | Patient (1) 48 hours  Patient (2) 32 hours  Patient (3) 48 hours  Patient (4) 8 hours | NR | Patient (1) liver and kidney injury non therapeutic  Patient (2) liver and kidney injury with coleperitoneum  Patient (3) liver injury with coleperitoneum  Patient (4) liver injury with hemoperitomeum |
| Zafar et al.^46^ | 3564 | 740 | 602 | 76.3% patients failed within  24 hours after admission | NR | NR |
| Hope et al.^47^ | 6 | 1 | 0 | Day 3 | Increased white blood count and fever | Nontherapeutic liver and splenic injury. Possible sealed gastric injury |
| Mnguni et al.^36^ | 133 | 0 | 0 | NA | NA | NA |
| Schnuriger et al.^67^ | 11 | 1 | 1 | 9 hours | NR | Colon injury |
| Fikry et al.^31^ | 38 | 7 | 7 | Patient (1) 3.5 hours  Patient (2) 3.5 hours  Patient (3) 4.5 hours  Patient (4) 3.0 hours  Patient (5) 5.0 hours  Patient (6) 4.8 hours  Patient (7) 11.2 hours | Worsening abdominal signs and symptoms | Patient (1) Grade V kidney injury  Patient (2) Spleen and diaphragm injury  Patient (3) small bowel injury  Patient (4) spleen, distal pancreas and stomach injury  Patient (5) stomach and diaphragm injury  Patient (6) stomach and liver injury  Patient (7) colon injury |
| Bjurlin et al.^32^ | 25 | 0 | 0 | NA | NA | NA |
| Navsaria et al.^33^ | 33 | 3 | 3 | NR | Patient (1) acute abdomen  Patient (2) increasing transfusion requirements  Patient (3) pyrexia, worsening respiratory function | Patient (1) colon injury, liver injury, diaphragm injury  Patient (2) Distal pancreatectomy, splenectomy, liver injury and diaphragm injury  Patient (3) nephrectomy for shattered kidney |
| Voelzke et al.^37^ | 51 | 2 | 2 | NR | Persistent bleeding | Nephrectomy (2) |
| Navsaria et al.^52^ | 63 | 5 | 5 | Patient (1) 8 hours  Patient (2) 12 hours  Patient (3) 48 hours  Patient (4) 48 hours  Patient (5) 72 hours | Patient (1) acute abdomen  Patient (2) increasing transfusion requirements  Patient (3) pyrexia, worsening respiratory function  Patient (4) pyrexia, worsening respiratory function  Patient (5) acute abdomen | Patient (1) colon injury, liver injury, diaphragm injury  Patient (2) Distal pancreatectomy, splenectomy, liver injury and diaphragm injury  Patient (3) nephrectomy, liver and diaphragm injury  Patient (4) liver and diaphragm injury  Patient (5) liver and diaphragm injury |
| Schmelzer et al.^48^ | 19 | 6 | 6 | NR | NR | NR |
| Chamisa et al.^53^ | 19 | 2 | 2 | NR | Peritonitis and pyrexia | Small bowel injury |
| DuBose et al.^65^ | 144 | 1 | 0 | Day 8 after admission | Hypotension | Non therapeutic liver injury |
| MacLeod et al.^44^ | 396 | 74 | 74 | From an hour to 18 hours after admission | NR | NR |
| Demetriades et al.^38^ | 39 | 3 | 3 | Patient (1) 41 hours  Patient (2) 41 hours  Patient (3) 19 hours | NR | Patient (1) splenic and colon injury  Patient (2) Grade III kidney injury and diaphragmatic injury  Patient (3) Grade III liver injury |
| Velmahos et al.^12^ | 103 | 6 | 2 | Patient (1) 121 minutes  Patient (2) 307 minutes | Peritonitis | Patient (1) colon and small bowel injury  Patient (2) colon injury  Negative laparotomy (4 patients) |
| Omoshoro_J et al.^54^ | 33 | 2 | 1 | Patient (1) Day 3 after admission  Patient (2) Day 7 after admission | NR | Patient (1) Bleeding from Grade III renal injury requiring right nephrectomy.  Patient (2) Necrotizing fasciitis with no intraabdominal involvement |
| Munera et al.^11^ | 36 | 1 | 0 | NR | CT findings of liver and right diaphragmatic injury | Hemoperitoneum |
| Velmahos et al.^39^ | 792 | 80 | 57 | NR | NR | Rectum or colon injuries (23 patients)  Small bowel (15 patients)  Liver (10 patients)  Spleen (9 patients)  Kidney (9 patients)  Stomach (7 patients)  Bladder (5 patients)  Ureter (3 patients)  Gallbladder (1 patient)  Duodenum (1 patient) |
| Demetriades et al.^55^ | 16 | 5 | 4 | From 36 to 72 hours | Peritonitis and/or falling haematocrit | Liver and renal injury developed ACS (1 patient)  Oozing controlled by suturing or hemostasis agents (4 patients) |
| Velmahos et al.^68^ | 4 | 0 | 0 | NA | NA | NA |
| Adesanya et al.^62^ | 14 | 2 | 1 | NR | NR | Small bowel injury |
| Velmahos et al.^43^ | 130 | 4 | 0 | Patient (1) 6 hours  Patient (2) 6 hours  Patient (3) 20 hours  Patient (4) 100 hours | Increased abdominal tenderness (4 patients)  Fever (1 patient) | All 4 non therapeutic |
| Demetriades et al.^56^ | 106 | 14 | 5 | Patient (1) 13.4 hours  Patient (2) 7 hours  Patient (3) 8 hours  Patient (4) 6 hours  Patient (5) 48 hours | Increasing abdominal tenderness (13 patients)  Evidence of continued bleeding- falling Hematocrit (1 patient) | Patient (1) colon injury  Patient (2) colon injury  Patient (3) colon injury  Patient (4) colon injury  Patient (5) liver & kidney injuries |
| Wessells et al.^34^ | 14 | 0 | 0 | NA | NA | NA |
| Chmielewski et al.^41^ | 18 | 1 | 0 | Day 3 after admission | Fever, tachycardia and abdominal pain | Hemoperitoneum |
| Renz et al.^42^ | 13 | 0 | 0 | NA | NA | NA |
| Demetriades et al.^57^ | 41 | 7 | 7 | From 4 hours to 4 days after admission | Development of abdominal signs | Colon injuries (3 patients)  Small bowel injuries (3 patients)  Liver injury (1 patients) |
| Muckart et al.^58^ | 22 | 0 | 0 | NA | NA | NA |
| McAlvanah et al.^59^ | 101 | 1 | NR | NR | NR | NR |
| Lowe et al.^60^ | 55 | 0 | 0 | NA | NA | NA |
| Taylor et al.^49^ | 8 | 0 | 0 | NA | NA | NA |
| Richter et al.^63^ | 13 | 3 | 0 | NR | NR | All 3 non-therapeutic |
| Ryzoff et al.^61^ | 17 | 0 | 0 | NA | NA | NA |

Abbreviations: NR= Not Reported, NA= Not Applicable, SNOM= Selective Nonoperative Management, CT= Computed Tomography. ACS=Abdominal Compartment Syndrome, WBC= White Blood Count
